
JOURNAL INFORMATION
==============================
NLM Title Abbreviation: ScientificWorldJournal
Journal ID: ScientificWorldJournal
NLM Journal ID: 101131163
Journal ID: TSWJ

The Scientific World Journal

ISSN: 2356-6140
EISSN: 1537-744X
Publisher: Wiley

ARTICLE INFORMATION
==============================
PMCID: PMC6084191
PMID: 30147577
DOI: 10.1100/tsw.2001.209
Article ID: 358328
Article version: 1
Subjects: Short Report

Conversion from a Type I to a Type II Death Receptor-Mediated Pathway through A Partial Inhibition of Caspase-8

Johnson Bryan W.
Cepero Enrique
Boise Lawrence H.
Department of Microbiology and Immunology, University of Miami School of Medicine, P.O. Box 016960 (R-138), Miami, FL, 33101, USA
Publication date: 2001
Electronic publication date: 2001 Nov 2
Volume: 1
First page: 98
Last page: 98
Copyright: Copyright © 2001 Bryan W. Johnson et al.
Copyright year: 2001
License: This is an open access article distributed under the Creative Commons Attribution License, which permits unrestricted use, distribution, and reproduction in any medium, provided the original work is properly cited.
License URL: https://creativecommons.org/licenses/by/3.0/

==============================
Miami *Nature Biotechnology* Short Reports
*TheScientificWorld* (2001) 1 (S3), 98SR
ISSN 1532-2246; DOI 10.1100/tsw.2001.209

### CONVERSION FROM A TYPE I TO A TYPE II DEATH RECEPTOR-MEDIATED PATHWAY THROUGH A PARTIAL INHIBITION OF CASPASE-8

Bryan W. Johnson, Enrique Cepero, and Lawrence H. Boise

Department of Microbiology and Immunology, University of Miami School of Medicine, P.O. Box 016960 (R-138), Miami, FL, 33101

**INTRODUCTION.** Bcl-2 is the prototypical member of a family of proteins which, when overexpressed in cells, are capable of either inhibiting or inducing apoptosis[1,2]. We have previously reported that Bcl-2 can cooperate with inhibition of caspase activation by zVAD-fmk to block TNFα-induced cell death in a Bcl-2 cleavage-independent fashion[3]. We now propose a multiple pathway model of TNFα-induced cell death that diverges at least at the level of caspase-8 activation. In this model, one mitochondria-independent (Type I) pathway is blocked by zVAD-fmk while the other mitochondria-dependent (Type II) pathway is blocked by Bcl-2[4].

**METHODS.** The murine pro-B cell line FL5.12 was cultured and transfections were performed as described[3]. For TNFα-induced cell death experiments, transfectants were incubated 6 hrs. at 37$^{o}$C in the presence of recombinant human TNFα (TNF; 2 ng/ml) and cycloheximide (CHX; 10 μg/ml). Where indicated, the caspase inhibitor zVAD-fmk (Enzyme Systems Products) was added at the indicated concentrations. For anti-Fas cell death experiments, Fas-transfected FL5.12 cells were incubated with 1 μg/ml Jo2 (Pharmingen) and the indicated concentrations of zVAD-fmk. Viability was determined by flow cytometry double staining with Propidium Iodide (PI) and AnnexinV-FITC. Western blotting was carried out as described[4]. DEVDase activity was determined by incubating $5x10^{5}$ cells with 10 μM DEVD-$G_1D_2$ (Oncoimmunin, Inc.) at 37$^{o}$C for 1 hour and analyzing samples by flow cytometry. The mean fluorescence intensity of the DEVD-$G_1D_2$-positive cells was calculated relative to the mean fluorescence intensity of DEVD-$G_1D_2$-positive Neo cells cultured in medium alone.

**RESULTS AND DISCUSSION.** Our data are most consistent with a model in which zVAD-fmk converts the death signal from a Type I to a Type II pathway through a partial inhibition of caspase-8, thereby allowing for protection by Bcl-2 or Bcl-$x_L$ at the level of the mitochondria[4]. We are currently in the process of determining whether these results represent a receptor-specific or cell-specific phenomenon. We have found the same cooperativity between Bcl-2 expression and zVAD-fmk in another pro-B cell line, Baf3, in response to TNFα treatment. Also, Bcl-2 lowers the amount of zVAD-fmk required to inhibit anti-Fas induced cell death in Fas-transfected FL5.12 cells. Interestingly, lower doses of zVAD-fmk were required to inhibit anti-Fas-induced cell death in vector control-transfected cells than that which was required to inhibit TNFα-induced cell death in the same cells. Differences in the way TNFR1 and Fas receptor complexes are formed may determine the sensitivity to caspase inhibition. Conversely, the difference may be due to our comparison of a physiologic ligand (TNFα) to an antibodymediated death stimulus (anti-Fas). Experiments to distinguish between these possibilities will be presented.

**REFERENCES.**

1. Adams, J.M. and Cory, S. (1998) Science 281, 1322-1326
2. Gross, A., McDonnell, J.M., and Korsmeyer, S.J. (1999) Genes Dev. 13, 1899-1911
3. Johnson, B.W. and Boise, L.H. (1999) J. Biol. Chem. 274, 18552-18558
4. Johnson, B.W., Cepero, E., and Boise, L.H. (2000) J. Biol. Chem. 275, 31546-31553
